# Research on Spatial Magnetic Field Distribution of Magnetic Fluids Based on Microstructure

**DOI:** 10.3390/ma17122994

**Published:** 2024-06-18

**Authors:** Bin Zhang, Yapeng Zhang

**Affiliations:** 1 Hubei Key Laboratory of Digital Textile Equipment, Wuhan Textile University, Wuhan 430200, China; xlth0124@163.com; 2 School of Mechanical Engineering and Automation, Wuhan Textile University, Wuhan 430200, China

**Keywords:** magnetic fluids, magnetic field distribution, Monte Carlo method, magnetic dipole theory

## Abstract

This study focuses on the spatial magnetic field distribution of magnetic fluids, an extraordinary class of fluids composed of magnetic nanoparticles (MNPs), employing the Monte Carlo method to simulate the microstructure of magnetic fluids under an external magnetic field. On that basis, a model was established through magnetic dipole theory to delve into the spatial magnetic field distribution of magnetic fluids. The findings reveal that the application of a magnetic field leads to the formation of chain-like structures within the magnetic fluids, resulting in inhomogeneous spatial magnetic field distribution. The size and concentration of MNPs are crucial determinants that significantly affect the microstructure of magnetic fluid and its spatial magnetic field distribution. Furthermore, environmental conditions such as external magnetic field strength or temperature can also regulate the positions of MNPs within magnetic fluids and the spatial magnetic field distribution of the magnetic fluids. These observations enrich the comprehension of the fundamental mechanisms of magnetic fluids and their response to diverse factors, advancing the growing comprehension on the characteristics and applications of these remarkable magnetic fluids.

## 1. Introduction

Magnetic nanoparticles are nanoparticles with magnetic properties that exhibit different properties at the nanoscale than at the macroscale, such as quantum size effect, surface effect, and superparamagnetism [1]. These magnetic nanoparticles are usually made into colloidal solutions, namely magnetic fluids. Magnetic fluid is considered a functional fluid as it demonstrates excellent functional characteristics in an applied magnetic field and exhibits specific physical properties under certain conditions. These fluids offer the fluidity of liquid solvents yet retain the magnetism of solid MNPs, making them sought-after magnetic media with diverse applications across industries and healthcare [2,3]. For example, the displacement of magnetic nanoparticles generated in an AC magnetic field can be used to destroy tumor cells [4]. Additionally, research has revealed that MNPs within magnetic fluids collectively magnetize under an external field, generating an induced field, giving rise to a macroscopic magnetic response observed from the magnetic fluid. Thus, altering the internal magnetic moment of MNPs with an alternate field can cause fluctuations in the magnetic flux of the receiver coil, enabling magnetic-electric signal detection and nanoimaging [5,6]. Magnetic fluids exhibit controllability under the regulation of external magnetic fields, making an in-depth understanding of their microstructure crucial.

With the rapid development of computer technology, molecular simulation technology has become an efficient and low-cost scientific research method. In the field of microstructure research of magnetic fluids, the main methods can be roughly divided into the Monte Carlo (MC) method and molecular dynamics (MD) method. The MD method is based on the interaction force between particles and simulates the microscopic process of complex systems by solving the motion equation of each particle. It is mainly used to study the dynamic behavior of magnetic fluids. For example, researchers used the MD method to study the dynamic properties of magnetic fluids under shear force [7]. The MC method, also known as the statistical simulation method, uses random sampling and probability statistics, without the need to solve complex mechanical calculations like the MD method. Through the MC method, researchers explored the microstructure formed by magnetic fluids under different magnetic fields. For instance, under a pulsed magnetic field, the magnetic fluids have been found to exhibit ellipsoidal structures [8]. In gradient magnetic fields, MNPs form a distribution gradient along the direction of the magnetic field gradient, and this gradient increases with increasing magnetic field gradient [9]. These studies have shown that the MC method can effectively and efficiently study the position distribution of nanoparticles in magnetic fluids. In previous studies, researchers have simulated the microstructure of magnetic fluids and conducted in-depth discussions on their optical and rheological properties, providing valuable insights into the behavioral mechanisms of these complex fluids. However, these studies often ignore the inherent magnetic properties of magnetic fluid as a magnetic medium, especially the induced magnetic field generated by magnetic fluid under the application of an external magnetic field, and the influence of the induced magnetic field on the external magnetic field. Therefore, investigating the magnetic properties of magnetic fluids from the perspective of the magnetization mechanism of MNPs presents a significant angle of consideration. MNPs have unique superparamagnetic properties and can respond quickly to an external magnetic field. Under the influence of an external magnetic field, the magnetic moment angle of the magnetic nanoparticles will follow the Boltzmann distribution along the direction of the external magnetic field [10]. In static or slowly varying magnetic fields, the magnetization process of magnetic nanoparticles follows the Langevin equation [11]. Moreover, under an external excitation magnetic field, the magnetized magnetic nanoparticles will generate an induced magnetic field, thereby affecting the spatial distribution of the magnetic field. For instance, in magnetic resonance imaging, magnetic nanoparticles are commonly employed as contrast agents, and their magnetization response under an applied excitation magnetic field impacts the uniformity of the surrounding magnetic field, consequently affecting the resonance signal [12]. Therefore, studying the interaction of magnetic nanoparticles with external magnetic fields is of great significance for expanding the application of magnetic nanoparticles in medicine.

This study investigates a magnetic field scenario by focusing on the microstructures of magnetic fluids simulated using the Monte Carlo method. Utilizing magnetic dipole theories, we construct an induced magnetic field approximation for these fluids, allowing us to determine their spatial magnetic field distribution. Furthermore, we examined how external variables like the imposed magnetic field strength and temperature, and critical nanoparticle properties such as particle size and concentration, impact the magnetic field distribution within the fluid space. These examinations enhance our comprehensive understanding of both the microstructural composition of magnetic fluids and the interplay among magnetic nanoparticles, external fields, and material attributes, which is crucial for advancing the applications of magnetic fluids.

## 2. Models and Methods

### 2.1. Monte Carlo Simulation

Magnetic fluids comprise magnetic nanoparticles, surfactants, and a base fluid, forming an equilibrium colloidal suspension. Consequently, their microstructure entails studying magnetic nanoparticle behavior. The magnetic nanoparticles undergo continuous Brownian motion, leading to the time-varying position distribution and relatively complex microstructure of magnetic fluids. To study this complex system, we consider employing the Monte Carlo method, which is a simulation technique based on probability and statistical theory. By generating many random numbers, the Monte Carlo method can simulate the occurrence process of events or the formation process of objects, thereby revealing the change patterns of events or the characteristics of objects. During the Brownian motion of the magnetic nanoparticles, the current state of the particles is determined solely by the previous state and is independent of the prior states, exhibiting the “memoryless” Markov property. Therefore, we can construct a suitable Monte Carlo Markov chain and describe the movement of magnetic nanoparticles within magnetic fluids based on the state transition probabilities. The state transition process of the Monte Carlo Markov chain requires substantial calculations, but this can be simplified using methods such as Metropolis sampling (also known as importance sampling). This sampling algorithm can efficiently achieve the steady-state distribution of the Markov chain.

Suppose the current state of the system is *x*(n), and after a change in the state of the particles in the system, the new state of the system is *x*(n + 1), and the system energy changes from *E*(n) to *E*(n + 1), then the energy change is Δ*E* = *E*(n + 1) − *E*(n) and the acceptance probability *p* for the change in the system state is the following:

(1) $$p=min\left\{1,\;exp\left(\frac{-\Delta E}{k_{b}T}\right)\right\},$$

where $k_{b}$ is the Boltzmann constant and T is the temperature of the system. For a system, the system always evolves toward a direction of entry reduction, or in other words, energy reduction. Therefore, after a change in the system state, if the system energy decreases, the energy change Δ*E* < 0, then the state change is considered reasonable; if the system energy remains unchanged or increases, the energy change Δ*E* ≥ 0, then the state change will be evaluated through probability calculations.

The Monte Carlo method simulates the microstructure of magnetofluids as follows:

1. Initialize the position and magnetic moment of the particles in the system.
2. Calculate the energy of the system in the current state *E*(n).
3. Select a particle at random and change its position or the direction of the magnetic moment.
4. Calculate the changed system energy *E*(n + 1) and the energy difference Δ*E*.
5. If Δ*E* < 0, adopt the changed state and proceed to the next state change.
6. If Δ*E* ≥ 0, a random number *R* ∈ [0, 1] is generated, and the transition probability *p* = exp(−Δ*E*/*k_b_T*) is calculated as follows:
  a. If *R* < *p*, adopt the new state, proceed to the next state change.
  b. If *R* ≥ *p*, reject the new state, proceed to the next state change.

Previous studies often assumed that the magnetic nanoparticles have a single particle diameter to form an ideal magnetic fluid. When studying the temperature sensitivity of MNPs, models based on a single particle size are often established [13]. In the study of the dynamics of magnetic fluids, ideal magnetic fluid assumptions are often used to simplify the model and analysis process [14]. However, in the actual preparation process, due to the influence of preparation techniques and conditions, it is difficult for the particle diameters of magnetic nanoparticles to achieve the ideal case of uniform particle diameters, and they typically follow a lognormal distribution [15,16].

Under a uniform magnetic field, the magnetic nanoparticles in the magnetic fluid are mainly influenced by three types of energies: the magnetic dipole–dipole interaction potential energy $u_{ij}^{m}$ between magnetic nanoparticles, the potential energy $u_{i}^{H}$ of magnetic nanoparticles under the magnetic field, and the surfactant repulsion potential energy $u_{ij}^{v}$ caused by the overlap of surfactant molecular layers. The various components of the energy for particles *i* and *j* are expressed as follows [17,18,19]:

(2) $$u_{i}^{H}=-{\left(\frac{d_{i}}{d_{0}}\right)}^{3}\mu_{0}m_{0}H\left(n_{i}\cdot h\right),$$

(3) $$u_{ij}^{m}={\left(\frac{d_{i}d_{j}}{{d_{0}}^{2}}\right)}^{3}\frac{\mu_{0}{m_{0}}^{2}}{4\pi {d_{0}}^{3}}\frac{{d_{0}}^{3}}{{r_{ij}}^{3}}\left[n_{i}\cdot n_{j}-3\left(n_{i}\cdot t_{ij}\right)\left(n_{j}\cdot t_{ij}\right)\right],$$

(4) $$u_{ij}^{v}=\frac{\lambda_{vi}k_{b}T}{2}\left\{2-\frac{2C_{i}}{t_{\delta i}}\ln\left(\frac{t_{\delta i}+1}{C_{i}}\right)-2\frac{C_{i}-1}{t_{\delta i}}\right\}+\frac{\lambda_{vj}k_{b}T}{2}\left\{2-\frac{2C_{j}}{t_{\delta i}}\ln\left(\frac{t_{\delta i}+d_{j}/d_{i}}{C_{j}}\right)-2\frac{C_{j}-d_{j}/d_{i}}{t_{\delta i}}\right\},$$

(5) $$C_{i}=\frac{{\left(1+t_{\delta i}\right)}^{2}-{\left(d_{j}/d_{i}+t_{\delta i}\right)}^{2}+4{r_{ij}}^{2}/{d_{i}}^{2}}{4r_{ij}/d_{i}},$$

(6) $$C_{j}=\frac{{\left(d_{j}/d_{i}+t_{\delta i}\right)}^{2}-{\left(1+t_{\delta i}\right)}^{2}+4{r_{ij}}^{2}/{d_{i}}^{2}}{4r_{ij}/d_{i}},$$

(7) $$\lambda_{vi}={\left(\frac{d_{i}}{d_{0}}\right)}^{2}\frac{\pi {d_{0}}^{2}n_{s}}{2},$$

(8) $$\lambda_{vi}={\left(\frac{d_{j}}{d_{0}}\right)}^{2}\frac{\pi {d_{0}}^{2}n_{s}}{2},$$

where $d_{0}$ is the mean particle diameter, while $d_{i}$ and $d_{j}$ represent the diameters of particles *i* and *j*, $m_{0}$ is the intrinsic magnetic moment of the particle, and $n_{i}$ and $n_{j}$ denote the vectors of the particle magnetic moments, with $n_{i}=m_{i}/m_{0}$, $n_{j}=m_{j}/m_{0}$; $\mu_{0}$ is the vacuum permeability; h is the unit vector of the applied magnetic field, with h=H/H ($H=\left|H\right|$) and $t_{ij}$ denoting the unit position vector from particle i to particle j given by $t_{ij}=r_{ij}/r_{ij}$ ($r_{ij}=\left|r_{ij}\right|$); $n_{s}$ is the number of surfactant molecules per unit surface area of the particle; and $t_{\delta i}$ is employed to describe the relationship between the surfactant thickness and article diameter, with $t_{\delta i}=2\delta /d_{i}$.

### 2.2. Spatial Magnetic Field of Magnetic Fluid

**Figure 1:**
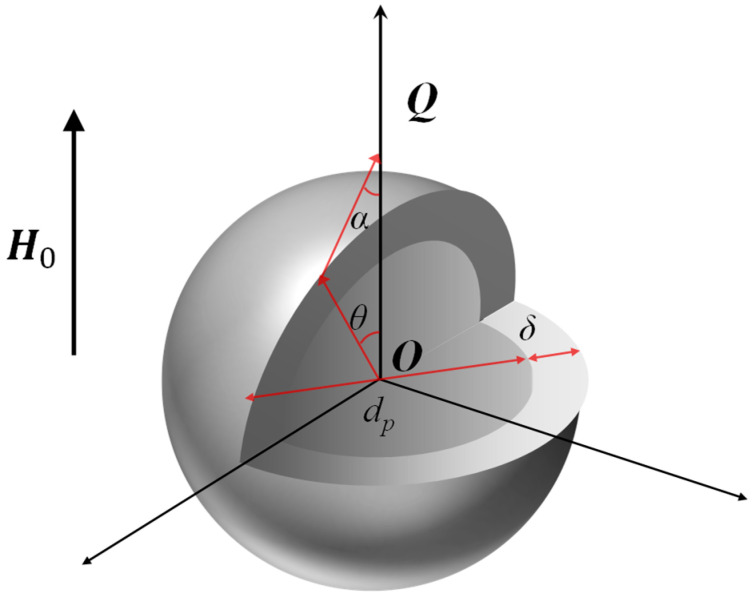
Magnetic nanoparticles in magnetic fields.

According to the magnetic dipole viewpoint, magnetic phenomena involve the presence of positive magnetic charges congregating at the N pole and negative magnetic charges congregating at the S pole, similar to the case of positive and negative electric charges. Since the existence of magnetic monopoles has not been conclusively demonstrated yet, a closed-loop current is typically employed to describe the physical model of a magnetic dipole. The model diagram of a spherical magnetic nanoparticle wrapped by a surfactant molecular layer is shown in Figure 1. The diameter of the magnetic nanoparticle is $d_{p}$, the thickness of the surfactant molecular layer is δ, and the overall radius is $a={(d}_{p}+2\delta )/2$. Assuming that magnetic nanoparticles have a single-domain structure, their magnetic moments can be considered fixed, and the particles only have uniaxial magnetic anisotropy, where the two easy magnetization directions are opposite [20]. Under the influence of an applied uniform magnetic field $H_{0}$, assume that the external permeability is $\mu_{1}$ and the internal permeability of the particle is $\mu_{2}$. With the center of the sphere as the origin, a spherical coordinate system is established with the direction of the applied magnetic field as the polar axis, and a symmetrical point Q on the polar axis is selected. To calculate the magnetic field strength at point Q, based on the boundary condition of equal magnetic field strengths inside and outside the sphere, the magnetic field strengths inside and outside the magnetic nanoparticle can be derived and represented by the following formulas [21]:

(9) $$p_{m}=\frac{4\pi \mu_{1}\left(\mu_{2}-\mu_{1}\right)a^{3}}{2\mu_{1}+\mu_{2}}H_{0},$$

(10) $$H_{i}=\frac{3\mu_{1}}{2\mu_{1}+\mu_{2}}H_{0},$$

(11) $$H_{e}=H_{0}+\frac{1}{4\pi \mu_{1}}\sum_{i=1}^{n}\left[\frac{3\left(p_{m}\cdot r\right)r}{r^{5}}-\frac{p_{m}}{r^{3}}\right],$$

where $H_{0}$ represents the strength of the external magnetic field, r is the distance from any point in space to the magnetic nanoparticle ($r=\left|r\right|$), $p_{m}$ is the magnetic dipole moment, $H_{i}$ is the strength of the internal magnetic field of the particle, and $H_{e}$ is the strength of the external magnetic field of the particle.

**Figure 2:**
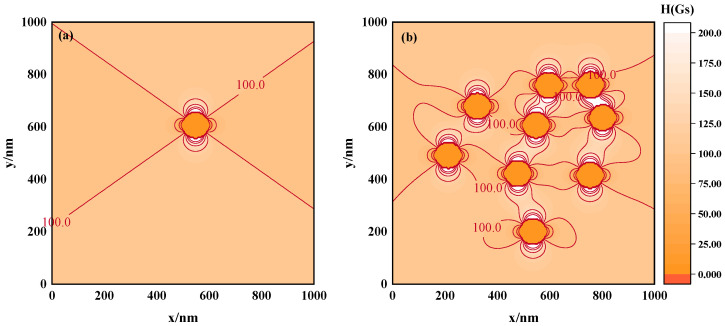
Equipotential diagrams of magnetic field strength. (**a**) Individual nanoparticle, (**b**) multiple nanoparticles.

**Figure 3:**
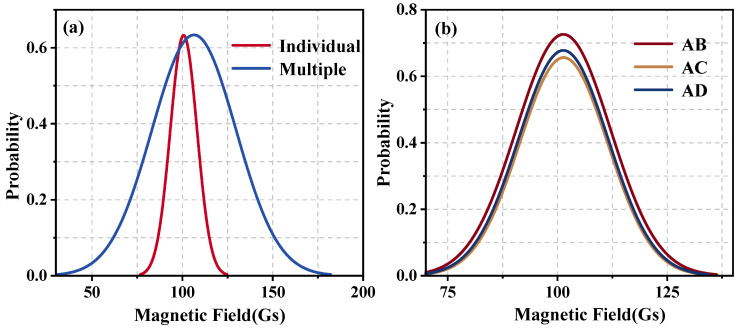
Magnetic field distribution curve. (**a**) Individual nanoparticle, (**b**) multiple nanoparticles.

In analyzing a single magnetic nanoparticle, we used mathematical formulations to evaluate the magnetic field strength at specific locations. Figure 2a shows the magnetic field strength equipotential diagram of a magnetic nanoparticle with a particle size of 100 nm under a uniform magnetic field of 100 Gs. The results demonstrate that the induced magnetic field produced after the magnetization of the magnetic nanoparticle leads to an inhomogeneous distribution of the magnetic field. In fact, the magnetic field intensity inside the magnetic nanoparticle is equal everywhere and is very small relative to the external magnetic field intensity, so the internal magnetic field intensity of the particles can be ignored. Based on this, we counted the frequency of the magnetic field strength and plotted a histogram of the magnetic field strength frequency distribution, thereby obtaining the spatial magnetic field distribution curve of the magnetic nanoparticle (Figure 3a).

According to the principle of magnetic field superposition, the total magnetic field at a given point in space is the vector sum of the magnetic fields generated by each individual magnetic field source at that location. Thus, the overall magnetic field of a magnetic fluid can be regarded as the result of the superposition of the magnetic fields produced by each constituent magnetic nanoparticle within the fluid. To illustrate this concept, several points in space and a system consisting of 9 MNPs were set up. The diameter of each particle was 100 nm. Under the interaction between particles and the external magnetic field, the position distribution of a random state was selected and the superimposed magnetic field strength of these points was calculated. The resulting equipotential diagram of the superimposed magnetic field strength is shown in Figure 2b. As evident from the figure, in the case of multiple nanoparticles, the spatial magnetic field presents an inhomogeneous distribution. Moreover, compared to the individual nanoparticle, the spatial field distribution has developed further. The curve presented in Figure 3a reflects the impact of the nanoparticles on the field homogeneity; the curve deviates from the external 100 Gs magnetic field, indicating that the external magnetic field is affected by the presence of a magnetic nanoparticle. Then, compared with a single particle, the center of the magnetic field distribution curve of multiple particles further deviates from 100 Gs, indicating that the distribution of the magnetic field in the multiple MNPs system is more complex. In addition, the full width at half maximum (FWHM) of the curve increases significantly, indicating that the distribution of the magnetic field has become more dispersed and inhomogeneous. These observations indicate that the presence of multiple nanoparticles intensifies the inhomogeneity of the magnetic field due to the intricate structure of the magnetic fluid.

**Figure 4:**
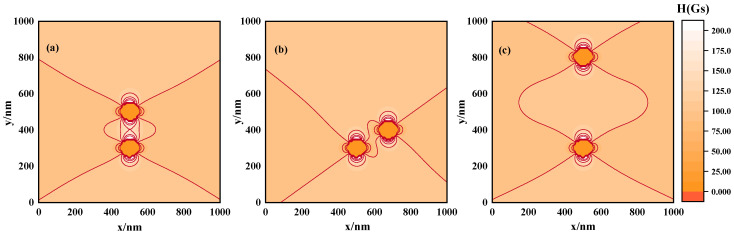
Equipotential diagrams of magnetic field strength of magnetic nanoparticles distributed at different locations. (**a**) Situation 1: a pair of particles arranged in the direction of the magnetic field, (**b**) Situation 2: a pair of particles that are offset at an angle along the direction of the magnetic field, (**c**) Situation 3: a pair of particles that are farther away in the direction of the magnetic field.

As illustrated in Figure 2b, the distribution of magnetic nanoparticles within magnetic fluids can significantly alter the spatial magnetic field profiles. Three distinct scenarios were studied to explore this phenomenon: aligned with the applied magnetic field (Figure 4a), placed at an incline (Figure 4b), and spaced farther apart (Figure 4c). These arrangements yield varied magnetic field profiles, as seen in Figure 3b and Table 1, based on our calculations and analyses. The findings indicate that decreasing particle separation intensifies magnetic field inhomogeneity, as evidenced by the comparison between Situation 1 and Situation 3. This suggests that denser structures result in more inhomogeneous field distributions. Moreover, tilting the particles relative to the field axis (Situation 2) without changing spacing still alters the field due to particle repositioning. These interpretations suggest that the ordered clustering of MNPs profoundly influences magnetostatic field inhomogeneity. The above results underscore the significant impact that the ordered structure formed in the magnetic fluid can have on the inhomogeneity of the magnetic field distribution.

### 2.3. Simulation Parameters

In this study, two-dimensional Monte Carlo simulations were used to investigate a two-dimensional system containing *N* MNPs. The particle size of the particles adopts lognormal distribution, with a mean value of $d_{0}$ and a standard deviation of σ = 0.2. The system boundary length $L\approx 60d_{0}$, the saturation magnetization of magnetic nanoparticles $M_{s}=4.168\times 10^{5}A/m$, the number of active agent molecules $n_{s}$ per unit surface area takes $10^{18}/m^{2}$, and the active agent thickness $\delta =0.15d_{0}$. A “periodic boundary condition” is introduced in the simulation process. When the movement of a particle exceeds the system boundary, it will enter the system again through the corresponding boundary. In molecular simulations, the cutoff radius $r_{coff}$ is usually set to simplify the calculation. In this paper, it refers to the maximum distance at which magnetic nanoparticles are considered to interact. Beyond this distance, the interaction is negligible, and $r_{coff}=10d_{0}$.

The specific simulation process was as follows: Initially, the N particles were arranged uniformly within the specified area, with minor disturbances introduced in their positioning and random magnetic moment vectors assigned. Currently, the state of the system is determined as the initial state of the particle. Subsequently, a random selection was made from the pool of particles, and their positions or magnetic moment direction were adjusted accordingly, resulting in a reformed status. This alteration was subjected to conditioning as stated in Section 2.1. The above process is regarded as a Monte Carlo step, and the above process is repeated. While attempting to modify the particle position or magnetic moment vector, the maximum displacement Δr of the particle is limited to $0.5d_{i}$, and the maximum angle Δθ of magnetic moment change was restricted to 10°.

The magnitude of the applied magnetic field, ambient temperature, particle diameter, and concentration of the magnetic nanoparticles significantly influence the magnitude of the magnetic moment and saturation magnetization of the nanoparticles, thereby subtly influencing the resultant spatial magnetic field pattern. To probe the impact of such modifying factors on the spatial magnetic field, the strategy of manipulating controllable variables during the simulation design was entertained. Amidst the aforementioned explicit parameters, a plethora of scenarios featuring varying particle diameters $d_{0}$, concentrations, magnetic field strengths *H*, and temperatures *T* were simulated. Solely, the concentration was manipulated by systematically altering the number of particles *N*. All other conditions remained unaltered, uniquely varying one variable and performing repetitive Monte Carlo simulations. Once the system energy approached equilibrium, the microstructure of the magnetic fluid was analyzed, and the frequency distribution of the magnetic field intensity was captured through measurements at multiple sampling points across the domain, yielding the spatial magnetic field profile of the magnetic fluid.

## 3. Results and Discussion

**Figure 5:**
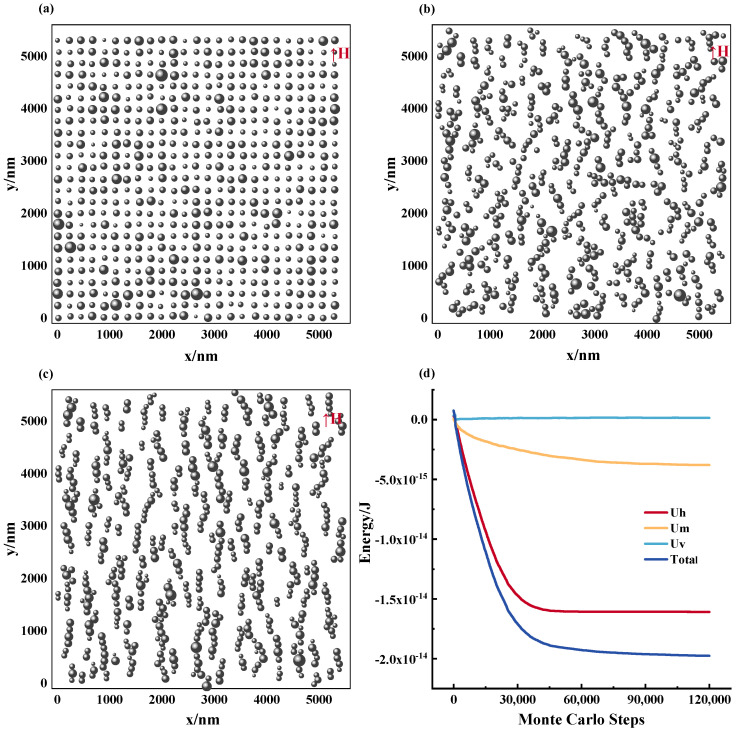
The formation process of the microstructure of magnetic fluids. (**a**) Step = 0, (**b**) step = 30,000, (**c**) step = 100,000, (**d**) the energy change process of the magnetic fluid system.

The characteristics of the microstructure of magnetic fluids evolve from its initial stage to a steady state, as illustrated in Figure 5a–c. These images demonstrate that the magnetic nanoparticles progressively align along the direction of the applied magnetic field, forming a chain-like configuration. This process is accompanied by the energy changes in the system, which are portrayed in Figure 5d; as the ordered structure is formed, the energy of the system gradually decreases and eventually reaches stability. The model inputs include initial nanoparticle dimension $d_{0}$ = 100 nm, particle count *N* fixed at 625, operating temperature *T* = 300 K, and applied magnetic field intensity *H* = 1000 Gs. For quantitative assessment of the magnetic nanoparticles’ distribution in the fluid, we utilize the contact coefficient $C_{con}$ and direction coefficient $C_{dir}$. The former denotes the ratio of chain-forming nanoparticles to the total number of particles, while the latter signifies the extent of their alignment along the magnetic field direction. Notably, particles were considered to be in contact if the interparticle distance $r_{ij}$ is less than contact radius $r_{c}$ ($r_{c}=d_{0}/10$), and they were considered to be aligned in the direction of the magnetic field if they were in contact and the spatial angle between the vectors of such particles < 30°. These quantitative analyses enabled the characterization of the chain-like structures formed by the magnetic nanoparticles and the evaluation of the level of orderly assembly within the fluid.

The results indicate that over time, the MNPs establish a discernible alignment along the magnetic field, ultimately leading to the formation of a chain-like structure. This behavior arises from the dominance of particle interactions over molecular thermal motion, promoting an organized arrangement of the MNPs [22]. In contrast to previously conducted research solely providing qualitative characterizations of the microstructural alterations in a magnetic fluid under the influence of an applied magnetic field, this study aims to extend the analysis by quantifying the impact of these microstructural modifications on the macroscopic magnetic field distribution properties.

### 3.1. Influence of the Particle Size on Magnetic Fluid Structure and Magnetic Field

**Figure 6:**
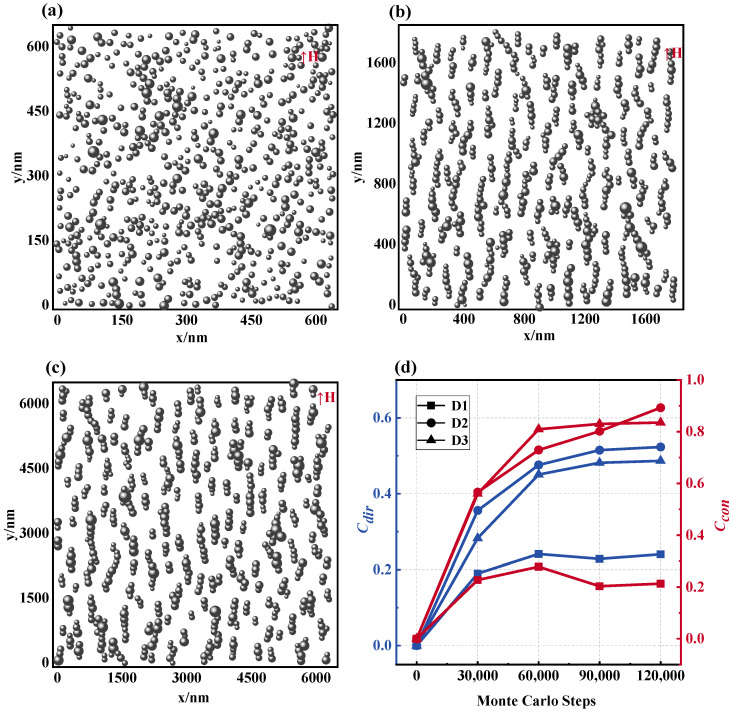
Microstructure of magnetic fluids at different particle sizes. (**a**) $d_{0}$ = 10 nm, (**b**) $d_{0}$ = 30 nm, (**c**) $d_{0}$ = 100 nm, (**d**) $C_{con}$ and $C_{dir}$ values of the microstructure of the magnetic fluid formed by different particle sizes: D1: $d_{0}$ = 10 nm, D2: $d_{0}$ = 30 nm, D3: $d_{0}$ = 100 nm.

This segment initially explores the microstructural evolution of magnetic fluids across varying particle sizes. Figure 6a–c depict the microstructure charts of the stabilized magnetic fluid at mean particle sizes $d_{0}$ = 10 nm, 30 nm, and 100 nm, under specific conditions of *N* = 625, *T* = 300 K, and *H* = 1000 Gs. The findings suggest that with smaller particle dimensions, the majority of the MNPs remain disordered and disorganized, with only a few forming short, curling chain configurations. As the particle size increases, progressively longer and denser chain structures emerge in the magnetic fluid, exhibiting a trend of interchain agglomeration. This correlation is reflected in the trajectory and values captured in the microstructure evolution plot of Figure 6d. Accordingly, an increase in particle size triggers a microstructural equilibrium, indicating that as changes in the contact coefficient $C_{con}$ and direction coefficient $C_{dir}$ plateau, a larger particle size yields a superior chain configuration. Notably, despite insignificant variations in the $C_{dir}$ values between the 30 nm and 100 nm particle sizes (D2 and D3), they outperform the 10 nm (D1) significantly, signifying that within a certain threshold, larger particle sizes render facilitation towards chain development along the magnetic field direction.

**Figure 7:**
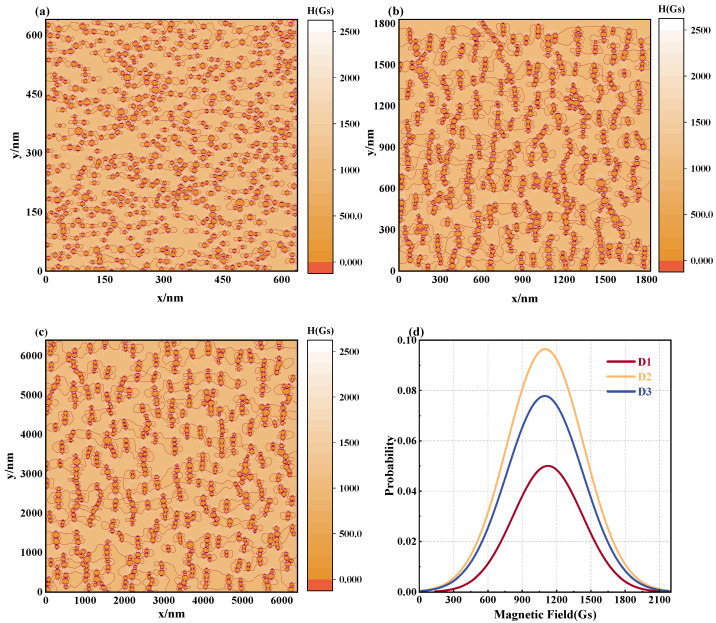
Effect of particle size on magnetic field distribution in magnetic fluid space: equipotential diagrams of the magnetic field of a magnetic fluid composed of different particle sizes for (**a**) $d_{0}$ = 10 nm, (**b**) $d_{0}$ = 30 nm, (**c**) $d_{0}$ = 100 nm, (**d**) magnetic field distribution curve: D1: $d_{0}$ = 10 nm, D2: $d_{0}$ = 30 nm, D3: $d_{0}$ = 100 nm.

Subsequently, to evaluate the spatial magnetic field distribution of the magnetic fluid, the equipotential chart of the magnetic field strength at distinct particle sizes is provided in Figure 7a–c. The observations convey that under diverse particle sizes, the spatial magnetic field experiences substantial alteration. Primarily, as the dimension of the MNPs increases, so does its saturation magnetization level. Therefore, under identical magnetic field influence, a more prominent induced magnetic field is produced among the MNPs, leading to an inhomogeneous field distribution enveloping the nanoparticles. Furthermore, the improved chain configuration created by a larger particle size results in a condensed within the chain region, manifesting a heightened magnetic field intensity surrounding the chain construct, creating a magnetic field augmentation zone. Figure 7d and Table 2 effectively describe the alterations in the magnetic field distribution. Specifically, with escalating the MNPs’ size, the acute displacement of the magnetic field distribution center in the magnetic fluid increases, accompanied by a surge in the FWHM of the magnetic field distribution. Nevertheless, beyond a particular limit, the spatial magnetic field heterogeneities of magnetic fluids begin to diminish. This is attributed to the extensive magnetic interactions among particles of exceedingly large particle size, posing a formidable ‘energy barrier’ and thus hindering the establishment and coalescence of the chain structure, consequently reducing the spatial magnetic field disparity. When nanoparticles aggregate, they form a lager cluster, which has stronger magnetic anisotropy, making the magnetic field distribution more complex. As anisotropy increases, the magnetic interactions between particles in the cluster may also become more intense, further significantly hindering the formation of chain structures [23].

### 3.2. Influence of the Number of Particles on Magnetic Fluid Structure and Magnetic Field

**Figure 8:**
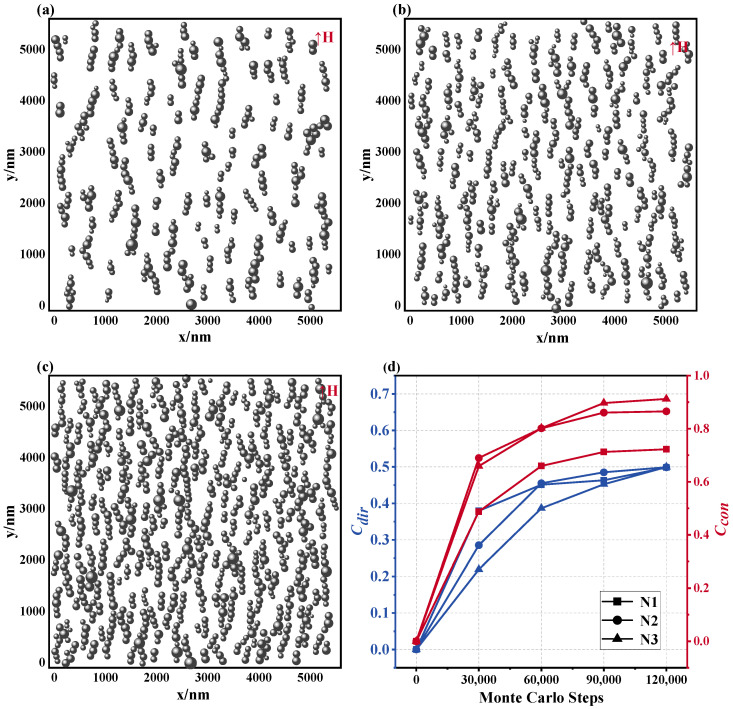
Microstructure of magnetic fluids with different numbers of magnetic nanoparticles. (**a**) *N* = 400, (**b**) *N* = 625, (**c**) *N* = 900, (**d**) $C_{con}$ and $C_{dir}$ values of the microstructure of the magnetic fluid formed by different particle numbers: N1: *N* = 400, N2: *N* = 625, N3: *N* = 900.

Figure 8a–c show that the microstructure of the magnetic fluid changes significantly when the number of particles N increases from 400 to 900, given that H = 1000 Gs, T = 300 K, and $d_{0}$ = 100 nm. In parallel, variations in $C_{con}$ and $C_{dir}$ are depicted in Figure 8d: As the number of particles increases, the value of $C_{con}$ also increases, which indicates that the rise in concentration is conducive to the formation of longer chain structures. This development is caused by the fact that an increase in concentration leads to a decrease in the space between the MNPs in their solvent, thereby increasing the likelihood of particle interactions and the rate of chain extension, ultimately resulting in the creation of longer chain structures. It is worth noting that in the three cases, the magnetic fluid has obtained similar $C_{dir}$ values in the stable state, which shows that the increase in the number of particles has no significant effect on the directivity of the chain structure.

**Figure 9:**
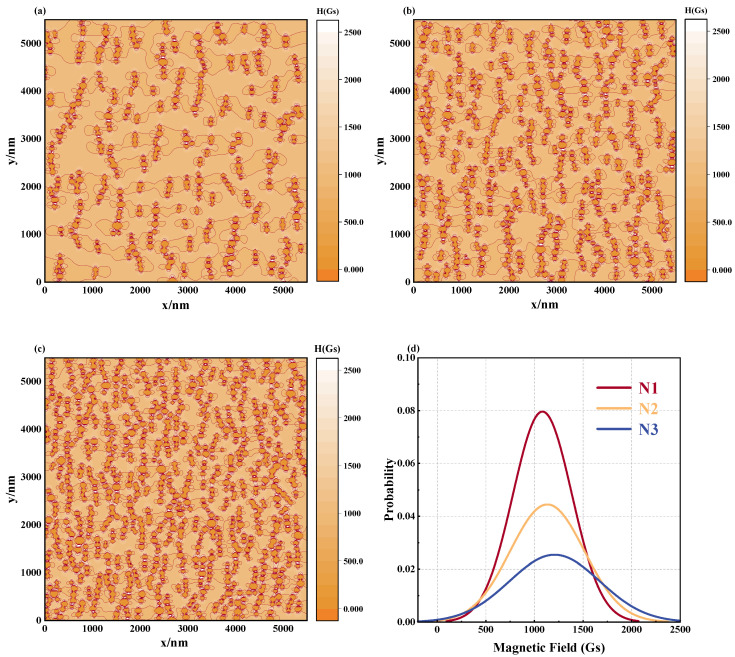
Effect of different particle numbers on magnetic field distribution in magnetic fluid space: equipotential diagrams of the magnetic field of a magnetic fluid composed of different particle sizes for (**a**) *N* = 400, (**b**) *N* = 625, (**c**) *N* = 900, (**d**) magnetic field distribution curve: N1: *N* = 400, N2: *N* = 625, N3: *N* = 900.

This transformation in the chain architecture directly impacts the magnetic field distribution profile of the magnetic fluids. As portrayed in Figure 9a–c, as the number of MNPs ascends, the equipotential diagram of the magnetic field intensity demonstrates increasingly inhomogeneous features. Examination of the magnetic field distribution graphs corresponding to Figure 9d reveals escalating central displacement and peak height. Extensive statistical analysis, outlined in Table 3, reveals that when the number of particles escalates from N1 to N3, the mean displacement of the magnetic field distribution increases from approximately 8.2% to 20.69%, accompanied by concurrent elevations in the FWHM, standard deviation, and coefficient of variation. The principal factors responsible for this alteration are the following: on the one hand, a substantial increase in the lengthwise chain structure within the paramagnetic medium due to an abundance of particles, subsequently enhancing the irregularity of the magnetic field distribution near the chain area; on the other hand, the narrowing of particle interstices triggers alterations in the localized magnetic field scope between diverse particles, consequently amplifying the disparity of the magnetic field dispersion around the magnetic nanoparticles. In nuclear magnetic resonance, magnetic nanoparticles change the resonance frequency of water molecules by affecting the homogeneity of the magnetic field, and this change can be captured in the spectrum [24]. The simulated curve results show that as the number of particles increases, the magnetic field distribution becomes more uneven, which is consistent with the experimental results. The relationship between the properties of magnetic nanoparticles and features of magnetic resonance provides support for magnetic resonance imaging.

### 3.3. Influence of the Magnetic Field Strength on Magnetic Fluid Structure and Magnetic Field

**Figure 10:**
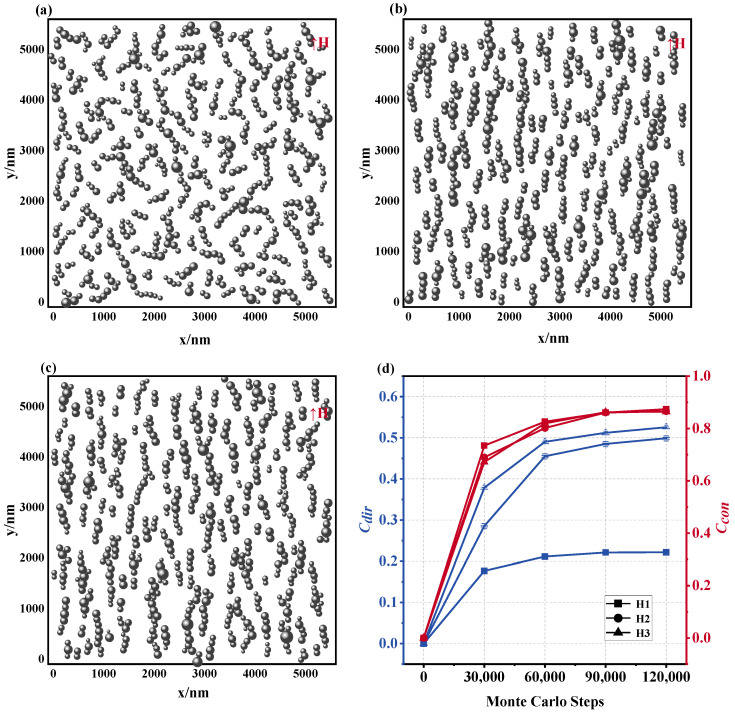
Microstructure of magnetic fluids at different magnetic field strengths. (**a**) *H* = 100 Gs, (**b**) *H* = 1000 Gs, (**c**) *H* = 5000 Gs, (**d**) $C_{con}$ and $C_{dir}$ values of the microstructure of the magnetic fluid formed by different magnetic field strength: H1: *H* = 100 Gs, H2: *H* = 1000 Gs, H3: *H* = 5000 Gs.

To investigate the effect of the magnetic field on the organization of the magnetic fluid, a set of parameters has been chosen, including a mean particle diameter of 100 nm, an operating temperature *T* = 300 K, and the total quantity of particles *N*. Refer to Figure 10a–c for the simulation outcomes. The results reveal that at lower magnetic field strengths, the magnetic fluid contains numerous short, curved chains, while fewer chains are formed along the magnetic field direction. Conversely, as the magnetic field strength increases, longer, more consistent chain-like formations begin to emerge within the magnetic fluid. This trend can be attributed to the increased magnetic stabilization offered by the increasingly powerful magnetic field superposition, leading to decreased thermal agitation and further fostering the formation of a more organized microstructural arrangement. Also, the variations of $C_{dir}$ seen in Figure 10d corroborate these assertions: the $C_{dir}$ value under a low magnetic field is significantly smaller than the $C_{dir}$ under a high magnetic field, and with the increase in magnetic field strength, the $C_{dir}$ value also increases. At the same time, $C_{con}$ does not show obvious differences in the three cases, which suggests that the magnetic field plays a decisive role in the directivity of the microstructure of the magnetic fluid, while its influence on the formation of the chain structure is not significant.

**Figure 11:**
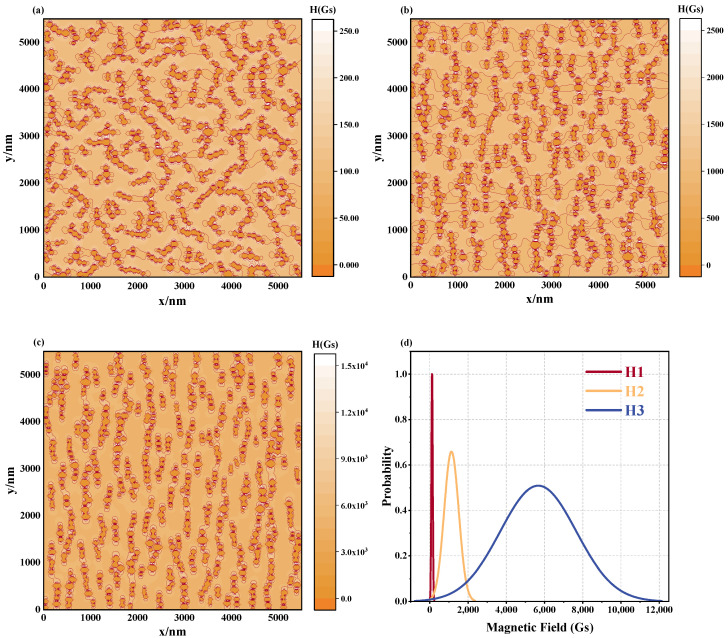
Effect of different magnetic fields on magnetic field distribution in magnetic fluid space: equipotential diagrams of the magnetic field of a magnetic fluid under different magnetic fields for (**a**) *H* = 100 Gs, (**b**) *H* = 1000 Gs, (**c**) *H* = 5000 Gs, (**d**) magnetic field distribution curve: H1: *H* = 100 Gs, H2: *H* = 1000 Gs, H3: *H* = 5000 Gs.

The equipotential charts in Figure 11a–c and the corresponding magnetic field distribution curve in Figure 11d further illustrate the impact of altering the magnitude of the external magnetic field. Altering the magnitude of the external magnetic field results in a more convoluted magnetic field distribution of the fluid, noticeable through an amplified center shift and broader areal variability depicted in Figure 11d. Given such considerable variance in magnetic field distribution under various conditions, the coefficient of variation serves as an effective metric for gauging the consistency of these distributions. As proved in Table 4, under the low strength magnetic field of 100 Gs, the coefficient of variation is 30.29%, exceeding that of the higher-field intensity configurations. This indicates that the local magnetic field distribution of the magnetic fluids exhibits excellent homogeneity under low field conditions. This difference can be analyzed from the microstructural perspective: at the low field strength (Figure 11a), the magnetic fluid formed short, curved chain-like structures, where the particles followed the field direction to a lesser degree, thereby weakening the effect of the external field and the corresponding local strong magnetic field areas. In addition, as the strength of the external magnetic field increases, the enhanced magnetization of the nanoparticles led to a more intense magnetization process, resulting in the increased inhomogeneity of the magnetic field around the particles. The reason for this difference can be analyzed from the perspective of microstructure; in Figure 11a, when the magnetic field strength is low, the magnetic fluid forms a short, curved chain-like structure. The particles in these short, curved chains follow the magnetic field direction to a lower degree, thereby weakening the effect of the external magnetic field on the particles and the local strong magnetic field area. In addition, the magnetization behavior of nanoparticles depends not only on the material properties of the nanoparticles themselves but also on the surrounding environment [25]. As the strength of the external magnetic field increases, the enhanced magnetization of the nanoparticles leads to a more intense magnetization process, resulting in the increased inhomogeneity of the magnetic field around the particles.

### 3.4. Influence of the Temperature on Magnetic Fluid Structure and Magnetic Field

Temperature plays a critical role as an environmental factor in the behavior of magnetic fluids, owing to the pronounced temperature sensitivity exhibited by the MNPs. The magnetic response of the MNPs demonstrates complex variations when subjected to different temperature measurement systems. In accordance with the Langevin model, numerous elements, including particle dimensions, saturation magnetization, and the applied magnetic field, contribute to controlling this temperature sensitivity [26]. To minimize the impact of temperature sensitivity, the simulations maintained a uniform particle diameter throughout our simulation, setting its value to $d_{0}$ = 30 nm and the total number of particles to *N* = 225. Additionally, the magnetic field strength was *H* = 500 Gs, while the experimental temperatures were *T* = 280 K, 300 K, and 320 K, respectively.

**Figure 12:**
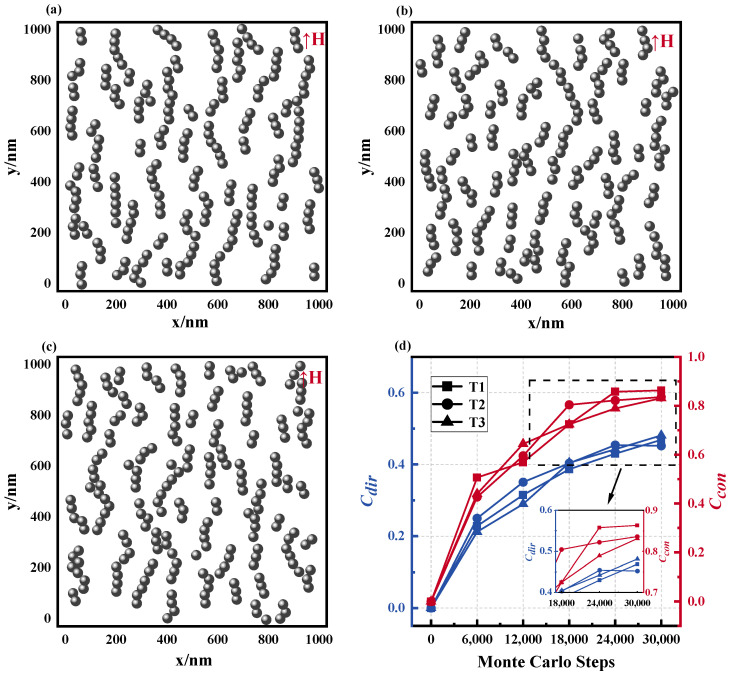
Microstructure of magnetic fluids at different temperature. (**a**) *T* = 280 K, (**b**) *T* = 300 K, (**c**) *T* = 320 K, (**d**) $C_{con}$ and $C_{dir}$ values of the microstructure of the magnetic fluid formed by different temperature: T1: *T* = 280 K, T2: *T* = 300 K, H3: *T* = 320 K.

**Figure 13:**
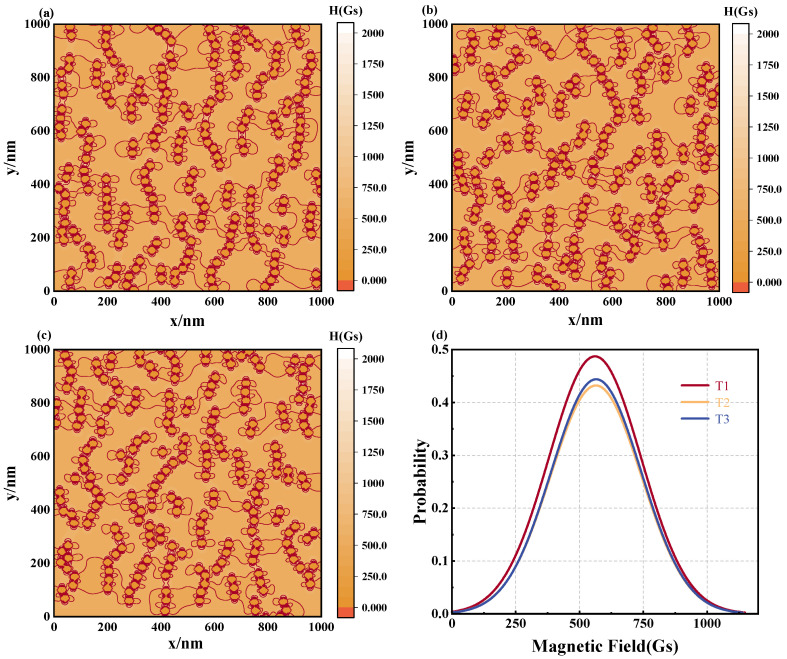
Effect of different temperature on magnetic field distribution in magnetic fluid space: equipotential diagrams of the magnetic field of a magnetic fluid composed of different temperature for (**a**) *T* = 280 K, (**b**) *T* = 300 K, (**c**) *T* = 320 K, (**d**) magnetic field distribution curve: T1: *T* = 280 K, T2: *T* = 300 K, H3: *T* = 320 K.

Figure 12 displays the microstructure of the magnetic fluid formed at various temperatures. As the temperature increases, the contact coefficient $C_{con}$ of the magnetic fluid gradually decreases when reaching stability, signifying the formation of a more disordered microstructure within the fluid. Examination of Figure 12a–c indicates that as the temperature ascends, the distribution of the MNPs within the magnetic fluid becomes increasingly confused and disordered. These findings suggest that the increase in temperature disrupts the formation of chain structures within the magnetic fluid, although the influence of temperature is relatively modest within the larger magnetic fluid system. To evaluate the distribution of the magnetic field, Figure 13a–c show the magnetic fluid spatial magnetic field equipotential diagram derived from calculations. Statistical analyses yield the magnetic field distribution curve illustrated in Figure 13d. The findings demonstrate that at a lower temperature of *T* = 280 K, the full width at half maximum of the magnetic field distribution curve markedly exceeds that of its elevated counterpart. This can be corroborated by the magnetic field distribution statistics listed in Table 5, where the standard deviation and the FWHM of T1 (at 280 K) exceed those of T2 (at 300 K) and T3 (at 320 K). Furthermore, while T2 exhibits minor increases over T3 in both standard deviation and the FWHM, they remain markedly smaller compared to that of T1. This suggests that increasing the temperature diminishes the irregularities in the magnetic field distribution of the magnetic fluid. This occurs primarily because higher temperatures suppress the magnetization response of nanoparticles, thereby reducing disparities in the local magnetic field distribution. Additionally, higher temperatures hinder the formation of more regular chains, further contributing to a reduction in the spatial magnetic field distribution disparity within the magnetic fluid.

Therefore, temperature, acting as a multifaceted environmental parameter, controls the overarching magnetic field distribution of the entire magnetic fluid system by influencing the magnetization characteristics of magnetic nanoparticles and shaping their microstructure. The magnetization response of magnetic nanoparticles changes with temperature. By detecting the magnetization response of these particles at different temperatures, an accurate measurement of the internal temperature of an organism can be achieved. A large amount of related research has been carried out in this field [27]. This multi-dimensional and multi-disciplinary thermo-response underscores the complexity of magnetic fluid dynamics and opens up novel avenues for comprehending and enhancing magnetic fluid efficiency, warranting further investigation.

## 4. Conclusions

This paper delves into the intricacies of the microstructure of magnetic fluid and its vital role in determining the spatial distribution of magnetic fields. Employing the Monte Carlo methodology, the microstructural properties of magnetic fluids are simulated, forming the foundation for a magnetic field model induced by the fluid, supplemented with statistical analyses to assess the homogeneity of the magnetic field within the system. The outcomes indicate that both the positional orientation and separation distance of the MNPs significantly impact the resultant spatial configuration of the magnetic field. When exposed to a uniform external field, these nanoparticles undergo a shift in position distribution, forming chain-like ordered structures that ultimately result in an inhomogeneous spatial magnetic field distribution. Particularly, an amplification in the particle dimension, concentration, or applied magnetic field improves the development of chain architecture, thereby heightening the disparity in the magnetic field configuration. Conversely, an escalation in the environment temperature disrupts chain architecture in the magnetic fluid, diminishing the inhomogeneous of the magnetic field distribution. Additionally, alterations in particle size, concentration, applied magnetic field, as well as temperature also manipulate the magnetization reaction of the MNPs, leading to shifts in the inhomogeneity of magnetic field distribution within the magnetic fluid environment. Magnetic field homogeneity is critical in nuclear magnetic resonance (NMR) applications, and the insights gained from this study offer promising directions for future applications. In NMR, the precision and accuracy of measurements depend heavily on the homogeneity of the magnetic field. The ability to fine-tune the microstructural properties of magnetic fluids to control the spatial distribution of the magnetic field can lead to significant advancements in NMR technology. For instance, enhanced magnetic field homogeneity can improve the resolution and sensitivity of NMR imaging and spectroscopy. Furthermore, the temperature sensitivity of MNPs can enable NMR systems that maintain optimal field homogeneity under varying operational conditions, enhancing the versatility and reliability of NMR instruments.

## Figures and Tables

**Table 1:** Statistics of magnetic field distribution of magnetic nanoparticles distributed at different locations.

	Mean (Gs)	Standard Deviation (Gs)	Coefficient of Variation (%)	FWHM (Gs)
Situation 1	101.358	10.74	10.596	25.29
Situation 2	101.409	10.04	9.903	23.65
Situation 3	101.347	10.22	10.086	24.07

**Table 2:** Statistics of the microstructure of magnetic fluids and the distribution of magnetic fields with different particle sizes.

	Microstructure		Magnetic Field Distribution			
*d*_0_ (nm)	$C_{con}$	$C_{dir}$	Mean (Gs)	Standard Deviation (Gs)	Coefficient of Variation (%)	FWHM (Gs)
10	0.21	0.25	1124.31	302.82	26.93	731.14
30	0.89	0.52	1101.11	334.81	30.41	784.48
100	0.84	0.49	1097.42	332.69	30.32	783.48

**Table 3:** Statistics of the microstructure of magnetic fluids and the distribution of magnetic fields with different particle numbers.

	Microstructure		Magnetic Field Distribution			
*N*	*C_con_*	*C_dir_*	Mean (Gs)	Standard Deviation (Gs)	Coefficient of Variation (%)	FWHM (Gs)
400	0.72	0.4983	1081.85	303.39	28.04	714.49
625	0.87	0.4991	1131.98	380.96	33.65	897.16
900	0.91	0.4994	1206.93	332.69	38.64	1098.16

**Table 4:** Statistics of the microstructure of magnetic fluids and the distribution of magnetic fields with different magnetic fields.

	Microstructure		Magnetic Field Distribution			
*H*(Gs)	$C_{con}$	$C_{dir}$	Mean (Gs)	Standard Deviation (Gs)	Coefficient of Variation (%)	FWHM (Gs)
100	0.874	0.222	116.29	35.22	30.29	82.95
1000	0.866	0.499	1131.98	380.96	33.66	897.16
5000	0.864	0.526	5673.73	1983.07	34.95	4670.12

**Table 5:** Statistics of the microstructure of magnetic fluids and the distribution of magnetic fields with different temperature.

	Microstructure		Magnetic Field Distribution			
*T* (K)	$C_{con}$	$C_{dir}$	Mean (Gs)	Standard Deviation (Gs)	Coefficient of Variation (%)	FWHM (Gs)
280	0.863	0.469	560.48	180.59	35.57	425.31
300	0.836	0.452	564.19	177.31	31.43	417.55
320	0.831	0.481	565.05	176.42	31.22	415.46

## Data Availability

Data are available from the corresponding author upon reasonable request.
